# Trichinellosis Outbreak Linked to Consumption of Privately Raised Raw Boar Meat — California, 2017

**DOI:** 10.15585/mmwr.mm6708a3

**Published:** 2018-03-02

**Authors:** Dustin Heaton, Sandra Huang, Rita Shiau, Shannon Casillas, Anne Straily, Li Kuo Kong, Valerie Ng, Viviana Petru

**Affiliations:** ^1^Acute Communicable Disease Section, Division of Communicable Disease Control and Prevention, Alameda County Public Health Department, Oakland, California; ^2^Parasitic Diseases Branch, Division of Parasitic Diseases and Malaria, CDC; ^3^Highland Hospital, Alameda Health System, Oakland, California; ^4^Department of Laboratory Medicine, University of California San Francisco, and Department of Laboratory Medicine & Pathology, Highland Hospital, Alameda Health System, Oakland, California; ^5^St. George’s University, St. George, Grenada.

On January 15, 2017, a hospital physician notified the Alameda County Public Health Department (ACPHD) in California of a patient with a suspected diagnosis of trichinellosis, a roundworm disease transmitted by the consumption of raw or undercooked meat containing *Trichinella spp.* larvae (*1*). A family member of the initial patient reported that at least three other friends and family members had been evaluated at area hospitals for fever, myalgia, abdominal pain, diarrhea, and vomiting. The patients had attended a celebration on December 28, 2016, at which several pork dishes were served, including larb, a traditional Laotian raw pork dish, leading the hospital physician to suspect a diagnosis of trichinellosis. Although the event hosts did not know the exact number of attendees, ACPHD identified 29 persons who attended the event and seven persons who did not attend the event, but consumed pork taken home from the event by attendees. The event hosts reported that the meat had come from a domesticated wild boar raised and slaughtered on their private family farm in northern California. ACPHD conducted a case investigation that included identification of additional cases, testing of leftover raw meat, and a retrospective cohort study to identify risk factors for infection.

## Investigation and Findings

Contact information for additional attendees and exposed persons was obtained during interviews with confirmed attendees. Reports of suspected diagnoses of trichinellosis among event attendees were requested from hospital infection prevention specialists, outpatient clinic providers, and local health jurisdictions where event attendees lived.

Exposure to *Trichinella* was defined as consumption of pork in which *Trichinella spiralis* larvae were identified. Thirty-six potentially exposed persons were identified, including 29 who attended the event and seven who consumed food taken home from the event by attendees. Among the potentially exposed persons, 20 (56%) were interviewed, 16 for whom professional language interpreters were used. Fourteen potentially exposed persons were not interviewed because contact information was unavailable, and two persons could not be reached. Clinical and exposure information from all 20 persons who were interviewed was collected using a structured questionnaire administered by telephone 28–92 days after the December 28 event. Medical records for patients with a suspected diagnosis of trichinellosis were requested from hospitals and outpatient providers and abstracted. In consultation with the California Department of Public Health and CDC, ACPHD recommended serologic testing for *Trichinella* for all persons with a suspected diagnosis of trichinellosis using a commercial laboratory’s enzyme-linked immunosorbent assay* to detect immunoglobulin G (IgG) directed against a *Trichinella* excretory-secretory antigen.

An illness that was clinically compatible with trichinellosis was defined as the occurrence of 1) myalgia and fever; or 2) periorbital edema; or 3) eosinophilia (≥6% eosinophils), with or without gastrointestinal symptoms (e.g., diarrhea, vomiting, or abdominal pain) in an attendee or someone who had consumed food brought home by an attendee. A probable case was defined as clinically compatible illness in a patient with exposure to *Trichinella*. Confirmed cases were defined as laboratory-confirmed *Trichinella* infection (i.e., a positive serologic test for *Trichinella* IgG antibodies) in a patient with history of exposure and clinically compatible illness.

Ten confirmed and two probable cases of trichinellosis were identified; 11 occurred in men. Eleven patients self-identified as Asian, and one identified as Asian and white. The median age was 58 years (range = 39–71 years). Onset dates ranged from December 28, 2016, to January 23, 2017. Nine patients were hospitalized, two of whom were admitted to the intensive care unit; nine had sepsis, seven had acute kidney injury, and two had gastrointestinal bleeding, one case of which was attributed to nonsteroidal antiinflammatory drug use. Eight patients had elevated peak creatine phosphokinase levels indicating skeletal muscle damage (median = 2,821 *μ*g/L; range = 566–25,467 [normal <200 *μ*g/L]), and seven had elevated peak lactic acid levels, which is an indicator of sepsis (median = 3.1 mmol/L; range = 2.3–5.3 [normal = 0.5–2.2 mmol/L]). Six had elevated peak troponin levels indicating damage to the myocardium (median = 0.76 *μ*g/L; range = 0.23–2.02 [normal <0.10 *μ*g/L]). Ten cases were confirmed by a positive *Trichinella* serological test; two patients were not tested (Table).

**TABLE:** Clinical characteristics of trichinellosis cases associated with consumption of raw boar meat (N = 12) — California, 2017

Characteristic	No. of patients (%)
**Sign/Symptom**	
Myalgia	12 (100)
Fever	11 (92)
Weakness	10 (83)
Chills	10 (83)
Diarrhea	9 (75)
Nausea/Vomiting	9 (75)
Abdominal pain	6 (50)
Cough	4 (33)
Shortness of breath	4 (33)
Periorbital edema	4 (33)
**Laboratory result**	
Reactive *Trichinella* immunoglobulin G	10 (83)
Elevated eosinophil percentage (≥6%)	10 (83)
Elevated creatine phosphokinase (>200 *μ*g/L)	8 (67)*
Elevated lactic acid (>2.2 mmol/L)	7 (58)*
Elevated troponins (≥0.1 *μ*g/L)	6 (50)*
**Treatment**	
Albendazole	11 (92)
Glucocorticoid	7 (58)
**Complication**	
Sepsis	9 (75)
Acute kidney injury	7 (58)
Gastrointestinal bleed	2 (17)
Death	0 (—)
**Highest level of care**	
Intensive care unit	2 (17)
Hospitalization	7 (58)
Emergency department	1 (8)
Outpatient	2 (17)

* Three patients had elevated creatine phosphokinase, lactic acid, and troponins.

Several event attendees had also assisted with food preparation. The three pork-containing dishes reported to have been served at the event included pork stew, grilled pork, and raw larb. Attendees were interviewed about preparation and consumption of the three pork dishes served at or taken home from the event, as well as consumption of any other pork-containing dishes served at the event and other sources of wild boar or bear meat. Attack rates and relative risks were calculated. Leftover raw pork from the implicated meal was obtained from the event hosts.

Larvae in an unstained touch preparation from the raw pork were verified as *Trichinella spp.* from a photomicroscopic image (Figure); samples were sent to CDC’s Division of Parasitic Diseases and Malaria diagnostic laboratory and identified as *Trichinella spiralis* through sequencing of the polymerase chain reaction–amplified ITS1-ITS2 region. Consumption of larb was significantly associated with trichinellosis, with an attack rate of 100% and a relative risk of 3.33 (95% confidence interval = 1.29–8.59). No other meat dishes were associated with an increased relative risk.

**FIGURE:**
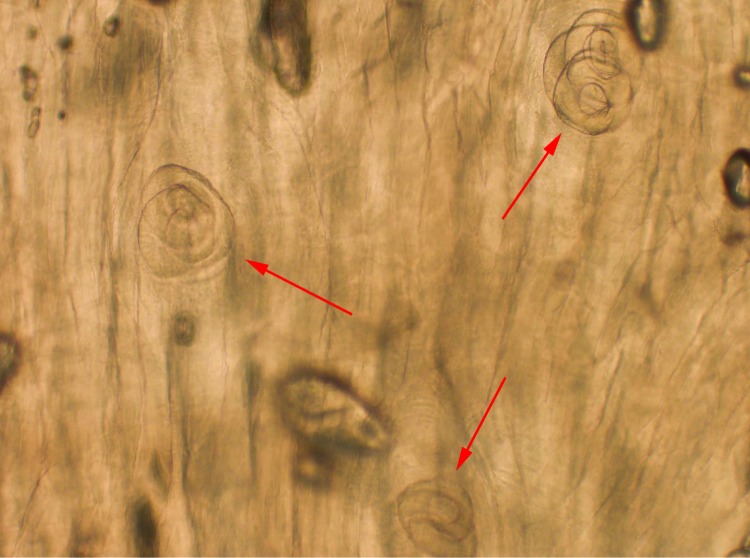
Microscopic image of *Trichinella spiralis* (arrows) encysted within implicated raw pork* — California, 2017 Photo/Valerie Ng, Department of Laboratory Medicine and Pathology, Highland Hospital, Alameda Health System * Unstained touch preparation, 100x magnification.

## Public Health Response

The caretaker of the source farm could not be reached, but the event host who owns the farm reported that the caretaker purchased the pig from a private farm at age 5 weeks, raised it in an outdoor, fenced pen, and slaughtered it with the farm owner at age 2.5 years. The farm owner stated there are several pigs being raised on the farm, and the swine are only given commercial feed and never cooked or uncooked meat, offal, or garbage. The farm owner denied any rodent infestation issues on the farm but did state that small animals such as chicks had occasionally gotten into the fenced pen and been eaten by the pigs, indicating that small mammals infected with *Trichinella* could have entered the pen and been consumed by the swine. The event host has slaughtered pigs and served the fresh raw pork dish at previous celebrations; no illnesses had been reported before this event. Health education regarding safe food handling practices and avoiding consumption of raw meat was provided during interviews with potentially exposed persons and patients. The host was educated about reducing the risk for trichinellosis when consuming pigs from his farm by freezing raw meat for 30 days and cooking meat to a minimum internal temperature of 160°F (71.1°C) to kill *Trichinella* larvae (*2*). Although the host did not indicate that he would employ these risk reduction techniques, he did state that he would not serve raw pork from pigs from his farm in the future. Some patients said they would no longer eat raw meat; one patient reported he would continue to eat raw meat from animals that he hunts, believing that raw meat confers strength.

## Discussion

Historically, most cases of trichinellosis were associated with the consumption of raw or undercooked *Trichinella*-infected pork (median = 360 cases reported to CDC per year during 1947–1956); however, largely owing to improvements in agricultural and food processing standards (*3*), many fewer cases are currently reported (median = 14.5 cases reported per year during 2006–2015) (*4*). Whereas trichinellosis is rare in the United States, it remains a public health threat, especially among populations that consume raw or undercooked wild game meat or pork from noncommercial sources (*5*). Recent outbreaks of trichinellosis have been associated with wild boar, bear, walrus, and unspecified pork (*4*,*6*). The outbreak described in this report was linked to consumption of a privately raised boar, yet surveillance data during 2008–2012 identified just one case of trichinellosis linked to the consumption of home-raised swine (*4*), suggesting that this might be an underrecognized risk factor for trichinellosis. Home-raised and home-slaughtered swine produced for personal consumption typically are not subject to the same safety and inspection standards as are commercially produced swine and might be outside the purview of inspections by the state agriculture department or animal health board. Home-raised swine with access to the outdoors are also at risk for acquiring other zoonotic parasites, including toxoplasmosis and *Ascaris suum* (large roundworm of pigs). Educating persons who raise swine for personal consumption about these safety concerns by public health or agriculture authorities might mitigate the risks.

Clinical disease associated with trichinellosis can be severe and might include sepsis, which has rarely been reported in the English-language scientific literature. This outbreak investigation indicates that high-risk meat preparation and consumption practices might be part of valued cultural traditions. Public health, agriculture, and wildlife authorities should strengthen efforts to provide culturally competent education about trichinellosis prevention to private farmers, hunters, and communities whose cultural practices include raw meat consumption.

**Summary**

**What is already known about this topic?**

Trichinellosis is a parasitic infection that can cause severe disease including sepsis. It is caused by the consumption of raw or undercooked meat containing *Trichinella spp.* larvae. Although strict agricultural and food processing standards have substantially reduced the prevalence of trichinellosis in the United States, persons who consume raw or undercooked wild game meat and pork from noncommercial sources remain at risk for the disease.

**What is added by this report?**

In January 2017, 12 cases of trichinellosis were reported among persons who attended an event on December 28, 2016, at which larb, a traditional Laotian raw pork dish, was served. The implicated pork came from a domesticated wild boar raised and slaughtered on a private farm in northern California; leftover samples were found to contain *Trichinella spiralis*. Nine infected persons were hospitalized with sepsis and seven had acute kidney injury.

**What are the implications for public health practice?**

Cultural practices that involve the consumption of raw meat might place certain groups at a higher risk for infection with *Trichinella* and other zoonotic parasites. Strengthening efforts by public health, agriculture, and wildlife authorities to provide culturally competent approaches to educating private farmers, hunters, and communities about trichinellosis prevention might reduce the risk for infection.

## Footnotes

* Gold Standard Diagnostics, testing performed at Focus Diagnostics, Inc., San Juan Capistrano, California.

## References

[1] American Public Health Association. Control of communicable diseases manual. 20th ed. Washington, DC: American Public Health Association; 2015.

[2] Food Safety and Inspection Service (FSIS), United States Department of Agriculture. FSIS compliance guideline for the prevention and control of trichinella and other parasitic hazards in pork and products containing pork. Washington, DC: Food Safety and Inspection Service, United States Department of Agriculture; 2016. https://www.fsis.usda.gov/wps/wcm/connect/2ca75475-3efd-4fa7-8f34-7393c245a1df/Trichinella-Compliance-Guide-03162016.pdf?MOD=AJPERES

[3] CDC. Parasites: trichinellosis (also known as trichinosis). Epidemiology & risk factors. Atlanta, GA: US Department of Health and Human Services, CDC; 2012. https://www.cdc.gov/parasites/trichinellosis/epi.html

[4] CDC. Surveillance for trichinellosis—United States, 2015 annual summary. Atlanta, GA: US Department of Health and Human Services, CDC, 2017. https://www.cdc.gov/parasites/trichinellosis/resources/trichinellosis_surveillance_summary_2015.pdf

[5] Wilson NO, Hall RL, Montgomery SP, Jones JL. Trichinellosis surveillance—United States, 2008–2012. MMWR Surveill Summ 2015;64(No. SS-01). 25590865

[6] Springer YP, Casillas S, Helfrich K, Two outbreaks of trichinellosis linked to consumption of walrus meat—Alaska, 2016–2017. MMWR Morb Mortal Wkly Rep 2017;66:692–6. 10.15585/mmwr.mm6626a3 28683055 PMC5726240

